# Display of a β-mannanase and a chitosanase on the cell surface of *Lactobacillus plantarum* towards the development of whole-cell biocatalysts

**DOI:** 10.1186/s12934-016-0570-z

**Published:** 2016-10-04

**Authors:** Hoang-Minh Nguyen, Geir Mathiesen, Elena Maria Stelzer, Mai Lan Pham, Katarzyna Kuczkowska, Alasdair Mackenzie, Jane W. Agger, Vincent G. H. Eijsink, Montarop Yamabhai, Clemens K. Peterbauer, Dietmar Haltrich, Thu-Ha Nguyen

**Affiliations:** 1 Food Biotechnology Laboratory, Department of Food Science and Technology, BOKU-University of Natural Resources and Life Sciences, Muthgasse 18, A-1190 Vienna, Austria; 2 BioToP the International Doctoral Programme on Biomolecular Technology of Proteins, Muthgasse 18, A-1190 Vienna, Austria; 3 Department of Biotechnology, DUT-Danang University of Technology, Nguyen Luong Bang, 54, Danang, Vietnam; 4 Department of Chemistry, Biotechnology and Food Science, Norwegian University of Life Sciences (NMBU), P.O. Box 5003, 1432 Ås, Norway; 5 Molecular Biotechnology Laboratory, School of Biotechnology, Suranaree University of Technology, Nakhon Ratchasima, Thailand

**Keywords:** Cell-surface display, Whole-cell biocatalyst, *Lactobacillus plantarum*, Mannanase, Chitosanase, Lipoprotein anchor, Cell wall anchor

## Abstract

**Background:**

*Lactobacillus plantarum* is considered as a potential cell factory because of its GRAS (generally recognized as safe) status and long history of use in food applications. Its possible applications include in situ delivery of proteins to a host, based on its ability to persist at mucosal surfaces of the human intestine, and the production of food-related enzymes. By displaying different enzymes on the surface of *L. plantarum* cells these could be used as whole-cell biocatalysts for the production of oligosaccharides. In this present study, we aimed to express and display a mannanase and a chitosanase on the cell surface of *L. plantarum*.

**Results:**

ManB, a mannanase from *Bacillus licheniformis* DSM13, and CsnA, a chitosanase from *Bacillus subtilis* ATCC 23857 were fused to different anchoring motifs of *L. plantarum* for covalent attachment to the cell surface, either via an N-terminal lipoprotein anchor (Lp_1261) or a C-terminal cell wall anchor (Lp_2578), and the resulting fusion proteins were expressed in *L. plantarum* WCFS1. The localization of the recombinant proteins on the bacterial cell surface was confirmed by flow cytometry and immunofluorescence microscopy. The highest mannanase and chitosanase activities obtained for displaying *L. plantarum* cells were 890 U and 1360 U g dry cell weight, respectively. In reactions with chitosan and galactomannans, *L. plantarum* CsnA- and ManB-displaying cells produced chito- and manno-oligosaccharides, respectively, as analyzed by high performance anion exchange chromatography (HPAEC) and mass spectrometry (MS). Surface-displayed ManB is able to break down galactomannan (LBG) into smaller manno-oligosaccharides, which can support growth of *L. plantarum.*

**Conclusion:**

This study shows that mannanolytic and chitinolytic enzymes can be anchored to the cell surface of *L. plantarum* in active forms. *L. plantarum* chitosanase- and mannanase-displaying cells should be of interest for the production of potentially ‘prebiotic’ oligosaccharides. This approach, with the enzyme of interest being displayed on the cell surface of a food-grade organism, may also be applied in production processes relevant for food industry.

## Background

The display of enzymatically active heterologous proteins on the bacterial cell surface is an attractive strategy for many biotechnological applications, such as development of whole-cell biocatalysts and biosensors [1]. One of the most attractive features of cell-surface display is that enzyme molecules are simultaneously synthesized and self-immobilized on the bacterial cell surface, and the living whole-cell biocatalyst can then be easily obtained from the cultivation [2–4]. Anchoring of a secreted enzyme to the bacterial cell wall enables the direct use of microbial cells as immobilized biocatalyst, simultaneously with or immediately after the fermentation step. This offers the known advantages of immobilization, such as reuse of enzyme, stabilisation, etc., while avoiding tedious enzyme purification steps, simplifying enzyme applications, and providing cost benefits [3, 5].

In principle, there are two different ways of anchoring a secreted protein to the bacterial surface: via covalent attachment to the cell membrane or the cell wall, or non-covalently via a protein domain that interacts strongly with components of the cell wall or the membrane. Both systems have been used in lactic acid bacteria (LAB), primarily in *Lactococcus lactis* and different lactobacilli [6, 7]. Covalent attachment to the membrane can be attained by lipoprotein anchors, which typically consist of an N-terminal signal peptide containing the lipobox motif in its C-terminal part. Following secretion of the protein by the Sec pathway, a diacylglycerol transferase covalently links a conserved cysteine in the lipobox to a phospholipid in the membrane, while the signal peptide is removed by a lipobox-specific peptidase. Anchoring of a recombinant protein to the cell membrane can thus be achieved by fusing a heterologous protein downstream of a suitable lipoprotein anchor. The use of lipoanchors for heterologous protein display has received relatively little attention in LAB, compared to other methods of surface display. Covalent anchoring to the cell wall can be achieved by employing the sortase (SrtA) pathway. Here, the secreted protein carries a C-terminal anchor containing the so-called LP×TG motif (LPQT×E in *L. plantarum* [8],) followed by a hydrophobic domain and a positively charged tail [9]. The hydrophobic domain and the charged tail keep the protein from being released into the medium, thereby allowing recognition of the LP×TG motif by a membrane-associated transpeptidase called sortase [9–11]. The sortase cleaves the peptide bond, e.g. between threonine and glycine in the LP×TG motif, and links the now C-terminal threonine of the surface protein to the pentapeptide cross bridge of peptidoglycan [6, 7, 10–13]. Two major differences exist between these two methods of covalent attachment: (1) The protein is attached either to the membrane or the cell wall, and therefore, sortase-mediated anchoring results in a more peripherally displayed protein. (2) The protein is attached to the cell surface via its N terminus with the lipobox approach, while the sortase-mediated cell wall anchor attaches the protein via its C terminus.

*Lactobacillus plantarum* WCFS1 is a well-studied member of the lactobacilli, which has been exploited as a host for cell-surface display of heterologous proteins, particularly for proteins with medical interest [1, 7, 14–17]. For example, cancer antigens have been expressed on the surface of *L. plantarum* using LP×TG anchoring, and it was shown that the recombinant bacteria induced specific immune responses in mice [17, 18]. In another study, an invasin protein, a virulence factor from the enteropathogenic bacterium *Yersinia pseudotuberculosis*, was covalently bound to the outer leaflet of the cell membrane using lipoprotein anchors and some of the resulting strains were powerful activators of NF-κB when interacting with monocytes [16]. These studies show the potential of anchoring functional proteins on the surface of *L. plantarum* using different anchoring methods.

In the present study, our aim was to display enzymes on the cell surface of *L. plantarum* and use the bacteria as whole-cell biocatalysts for the production of prebiotic oligosaccharides. For that purpose, we expressed and displayed a mannanase from *Bacillus licheniformis* DSM13 (ATCC 14580) and a chitosanase from *Bacillus subtilis* 168 (ATCC 23857) on the cell surface of *L. plantarum* WCFS1. Both enzymes are of interest for food and biotechnological applications. Mannanases (EC 3.2.1.78) release β-1,4-manno-oligosaccharides (MOS) from mannans, whereas chitosanases (EC 3.2.1.132) release chito-oligosaccharides (CHOS) from chitosans. The immobilization of active mannanase or chitosanase through cell-surface display could result in safe, stable food-grade biocatalysts that can be used in the production of MOS and CHOS in efficient and “food-grade” processes.

## Results

### Surface display of ManB and CnsA in *Lactobacillus plantarum*

**Fig. 1:**
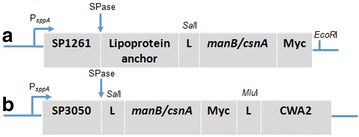
Schematic overview of the expression cassette for N-terminal lipoprotein anchoring (**a**) and C-terminal cell wall anchoring (**b**) of mannanase (ManB) and chitosanase (CsnA) in *L. plantarum*. The vectors are derivatives of previously described plasmids [16, 17, 31]. The inserts containing the *man*B or *csn*A sequence fused with a Myc tag for protein detection. All parts are easily interchangeable using the indicated linker (L) restriction sites (*Sal*I and *EcoR*I or *Mlu*I). See text for more details

**Fig. 2:**
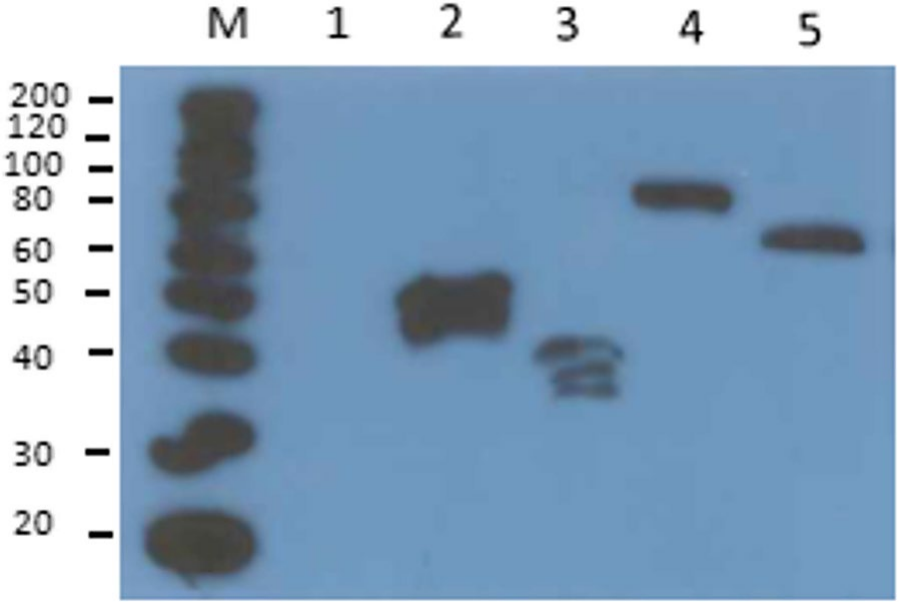
Western blot analysis of cell-free extracts from transformed and induced *Lactobacillus* cells harboring various expression vectors. (1) pEV, negative control; (2) pSIP_1261ManB (lipoprotein anchor; predicted protein size of 51 kDa); (3) pSIP_1261CsnA (lipoprotein anchor; predicted protein size of 37 kDa); (4) pSIP_ManBcwa2 (cell wall anchor; predicted protein size of 62 kDa); (5) pSIP_CsnAcwa2 (cell wall anchor; predicated protein size of 51 kDa). Lane M indicates a molecular mass marker

**Fig. 3:**
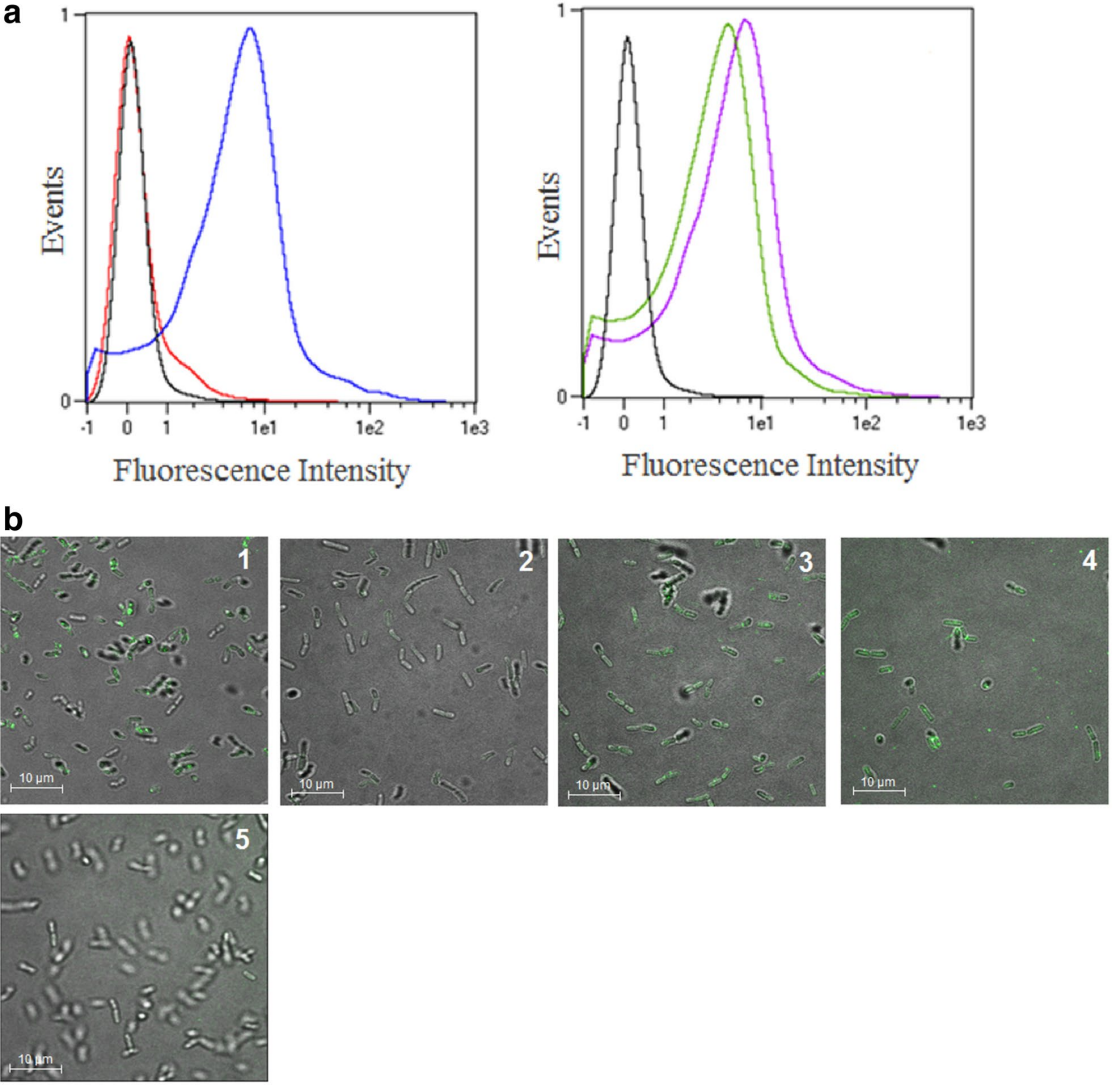
Surface localization of ManB and CsnA in *L. plantarum* cells. The panels show representative flow cytometric (**a**) and microscopic (**b**) analyses of *L. plantarum* cells harboring plasmids designed for cell-surface display of ManB and CsnA. The *L. plantarum* strains harboring ManB- or CsnA-encoding plasmids are denoted by* different colors* in the flow cytometry histograms (**a**) and different numbers in the micrographs (**b**): pSIP_1261ManB (*blue*, 1), pSIP_1261CsnA (*red*, 2), pSIP_ManBcwa2 (*green*, 3), pSIP_CsnAcwa2 (*purple*, 4). *L. plantarum* harboring an empty vector was used as negative control pEV (*black*, 5)

To display mannanase (ManB) and chitosanase (CnsA) on the surface of *L. plantarum* two anchors were exploited. The enzymes were N-terminally anchored to the cell membrane using a 75 residue lipoprotein anchoring sequence derived from the Lp_1261 protein of *L. plantarum* [16] (Fig. 1). In addition, to achieve cell wall anchoring, the enzymes were fused to an LP×TG anchor derived from the Lp_2578 protein sequence, also known as “cwa2” [17]. For immunodetection of the proteins, a Myc-tag was fused to the enzyme sequences as shown in Fig. 1. To test the expression of the target enzymes, the cells were harvested 2 h after induction and Western blot analysis of the crude, cell-free extracts was performed using anti-Myc antibodies for detection. Figure 2 shows that all four recombinant bacteria produced the expected proteins. Target proteins with cell wall anchor showed slightly higher size than expected, which is most likely a result of the presence of cell wall fragments associated with the target protein. The protein extracts from strains harbouring pSIP_1261ManB and pSIP_1261CsnA showed additional bands of smaller mass, which are most likely representing processed and unprocessed proteins. Mass differences such as those observed here are commonly reported in literature [19–21]. The discrepancy in size may be due to binding to peptidoglycans for LP×TG anchors (larger size than expected, multiple bands) and incomplete processing (multiple bands) or proteolysis (multiple bands) for lipo-anchors. The anchored proteins are likely to contain peptidoglycan fragments of various sizes, which explain a certain degree of heterogeneity. Flow cytometry confirmed surface display of the target enzymes in three of the four recombinant bacteria (pSIP_1261ManB, pSIP_ManBcwa2, and pSIP_CsnAcwa2; Fig. 3a) Even though CsnA production was clearly shown by Western blotting for the strain carrying pSIP_1261CsnA (Fig. 2), we could not observe a shift in the fluorescence signal for this strain compared to the control strain (Fig. 3a). Immunofluorescence microscopy confirmed surface localization of the Myc-tag in strains carrying pSIP_1261ManB, pSIP_ManBcwa2, pSIP_CsnAcwa2, while again no signal was obtained with pSIP_1261CsnA (Fig. 3b). Hence, it was confirmed that the cell wall anchor mediates surface localization of both ManB and CsnA, whereas the lipo-anchor mediates surface expression of ManB.

### Enzymatic activity and stability of ManB and CnsA-displaying cells

**Fig. 4:**
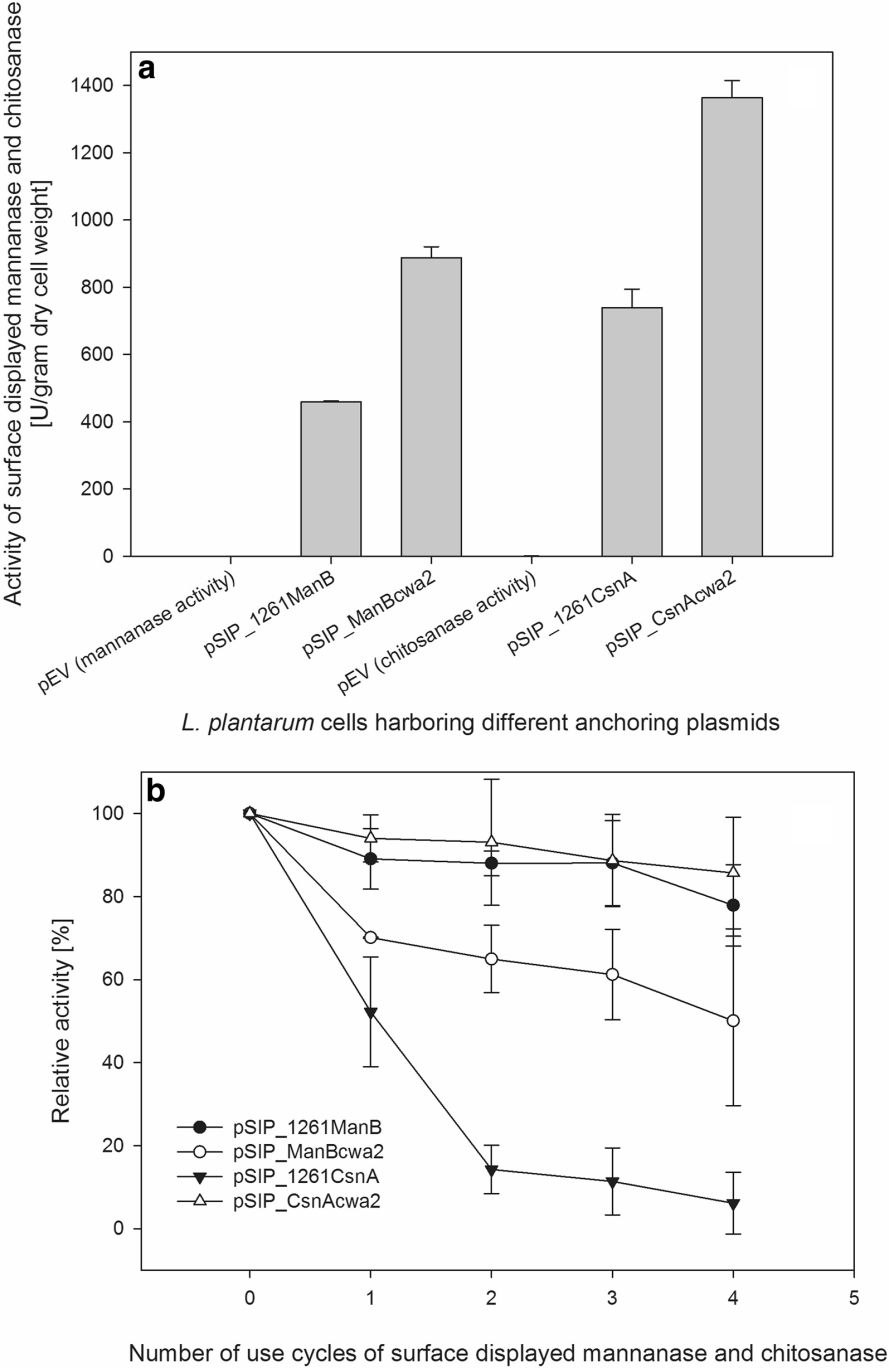
Enzyme activity of ManB- and CsnA-displaying cells. **a** Activities of freshly harvested cells with the *left* three and *right* three *bars* respresenting mannanase and chitosanase activity, respectively. *L. plantarum* harboring the empty vector, pEV, was used as negative control in both cases. **b** Results of repeated activity measurements, with 0 indicating freshly harvested cells, while 1, 2, 3, 4 indicated the number of repetitions. In between the activity measurements the cells were washed with PBS as described in “Methods” section

To test the functionality of the surface-displayed enzymes, we measured the enzyme activities of living recombinant bacteria. The highest enzymatic activities of ManB or CnsA-displaying cells were 890 and 1360 U per g of dry cell weight, respectively (Fig. 4a), which were obtained with the strains carrying plasmids for cell wall anchoring, pSIP_ManBcwa2 and pSIP_CsnAcwa2, respectively. The mannanase activity of the strain harboring the plasmid pSIP_1261ManB was determined to be ~460 U/g dry cell weight (Fig. 4a). Interestingly, significant chitosanase activity of ~740 U/g dry cell weight was found for the strain carrying pSIP_1261CsnA (lipo-anchored CsnA), even though surface localization of CsnA could not be confirmed by both flow cytometry or immmunofluorescence microscopy.

One advantage of immobilizing enzymes on the surface of bacteria is that they can easily be separated from the culture medium and reused. To test the stability of ManB and CsnA-displaying cells, we measured the enzyme activity during four repeated cycles with a washing step between the cycles to remove proteins released from lysed cells. The enzymatic activities of *Lactobacillus* cells harboring pSIP_CsnAcwa2 and pSIP_1261ManB decreased slightly as indicated by activity losses of ~14 and 22 %, respectively, after four assay/washing cycles, confirming that these enzyme-displaying cells can be reused for several rounds of biocatalysis at 37 °C (Fig. 4b). Significant losses in enzymatic activity were observed for cells harboring pSIP_ManBcwa2 and pSIP_1261CsnA, with only 70 and 15 % of the initial mannanase and chitosanase activity being retained after the second cycle, respectively. After the fourth cycle, 90 % of chitosanase activity and 50 % of mannanase activity were lost from cells carrying pSIP_1261CsnA and pSIP_ManBcwa2, respectively (Fig. 4b).

### Formation and analysis of oligosaccharides

**Fig. 5:**
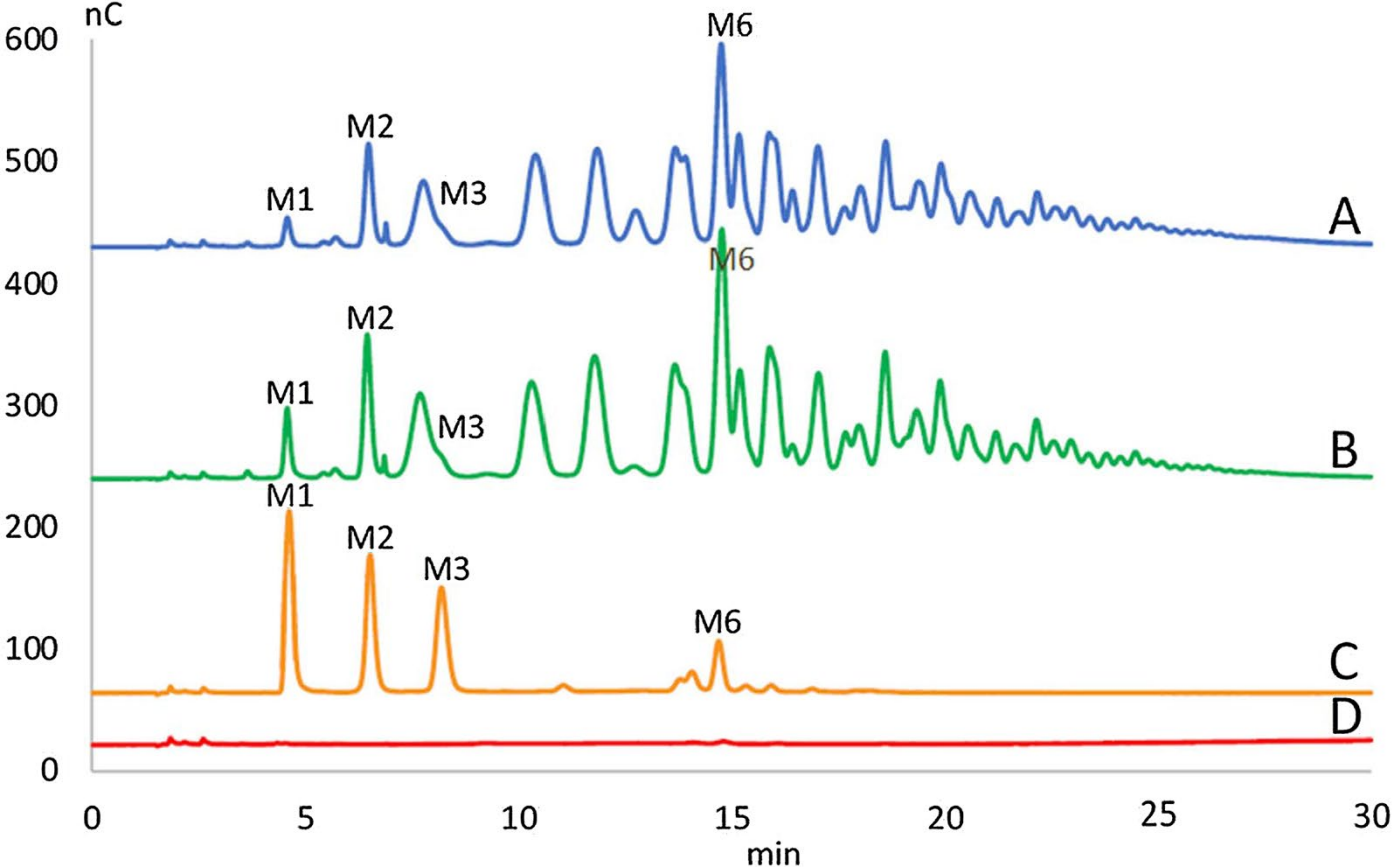
Formation of manno-oligosaccharides (MOS) from LBG by mannanase-displaying *L. plantarum* cells. The picture shows HPAEC chromatograms of supernatants from the activity assay displayed in Fig. 4 (freshly harvested cells). (A) pSIP_ManBcwa2; (B) pSIP_1261ManB; (C) Standards: mannose, M1, mannobiose, M2, mannotriose, M3, mannohexaose, M6; (D) empty vector, pEV as negative control. Note that degradation of LBG galactomannan by a mannanase is expected to yield a wide variety of products (for which there are no standards), as is indeed observed in traces (A) and (B)

**Fig. 6:**
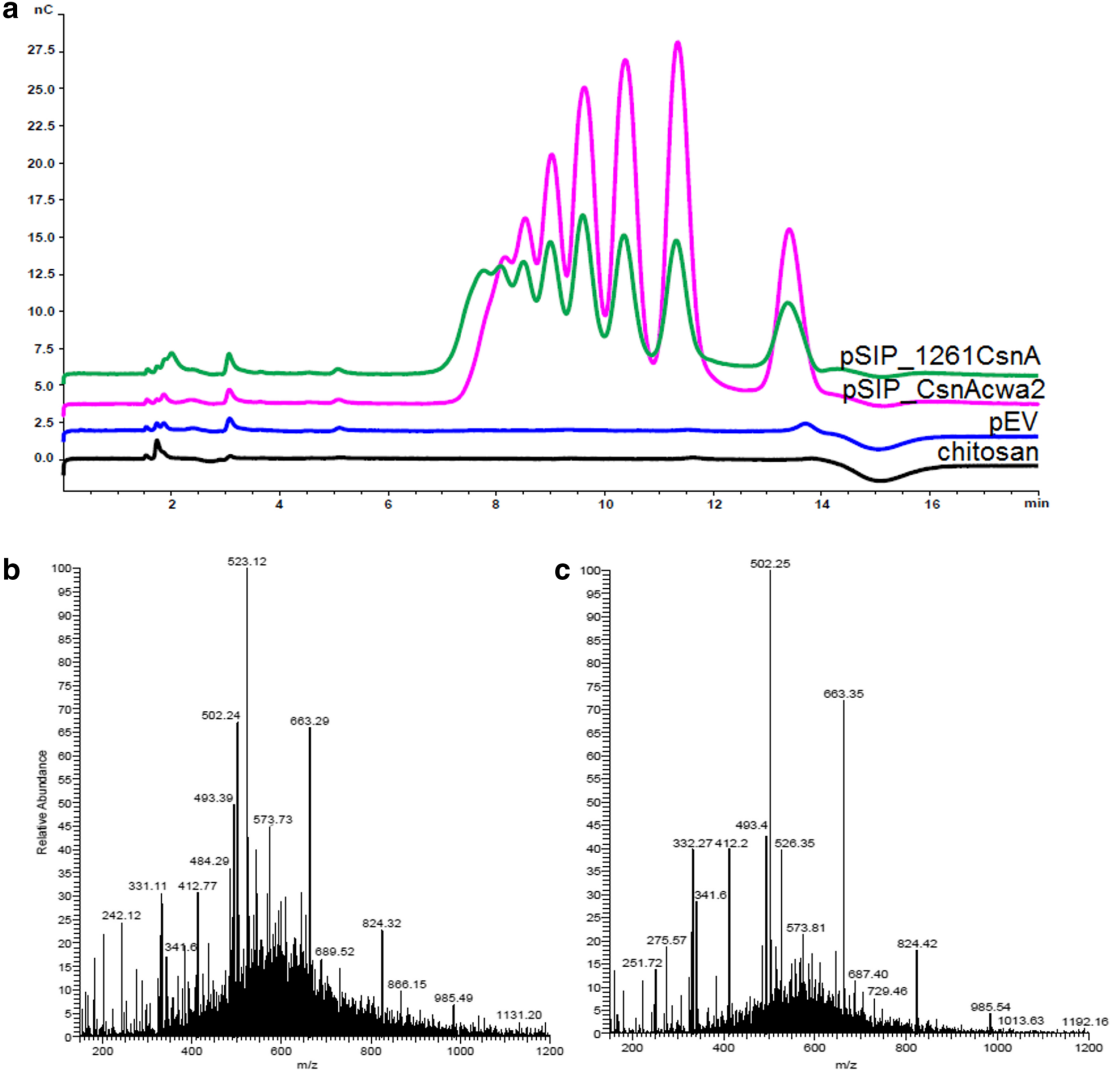
HPAEC chromatogram (**a**) and direct infusion ESI–MS analysis (**b**, **c**) of deacetylated chito-oligosaccharides (CHOS) obtained upon incubating *L. plantarum* cells harboring pSIP_1261CsnA (**b**) or pSIP_CsnAcwa2 (**c**) with chitosan. The HPAEC chromatograms include a negative control (pEV) and a substrate blank containing chitosan without addition of cells. Standards for deacetylated oligomers are not easily accessible. It is known from earlier work [37] that chitosan oligomers elute between 7 and 14 min, as is observed; the presence of such oligomers was confirmed by MS analysis. The MS-spectra show deacetylated CHOS from DP 2 to DP 6 {[M + H]^+^: *m/z* 341 (DP2), 502 (DP3), 663 (DP4), 824 (DP5), 985 (DP6); and double charged species of the same compounds; [M + 2H]^2+^: *m/z* 251 (DP3), 332 (DP4), 412 (DP5), 493 (DP6)}

The conversion of locus bean gum (LBG) to manno-oligosaccharides (MOS) by ManB-displaying cells was analyzed by high performance anion exchange chromatography (HPAEC) of supernatants from the enzyme activity assay, which confirmed the presence of a range of different MOS in the reaction mixtures already after 5 min of catalysis at 37 °C (Fig. 5). The conversion of chitosan to chito-oligosaccharides (CHOS) by CsnA-displaying cells was analyzed by HPAEC and direct infusion ESI–MS, and formation of deacetylated chito-oligosaccharides ranging from DP2 to DP6 was indeed observed (Fig. 6). Altogether, the results described so far indicate that ManB and CsnA displayed on *L. plantarum* cells are catalytically active and can convert their polymeric substrates into oligosaccharides.

### Growth of ManB-displaying *Lactobacillus plantarum* cells on LBG

**Fig. 7:**
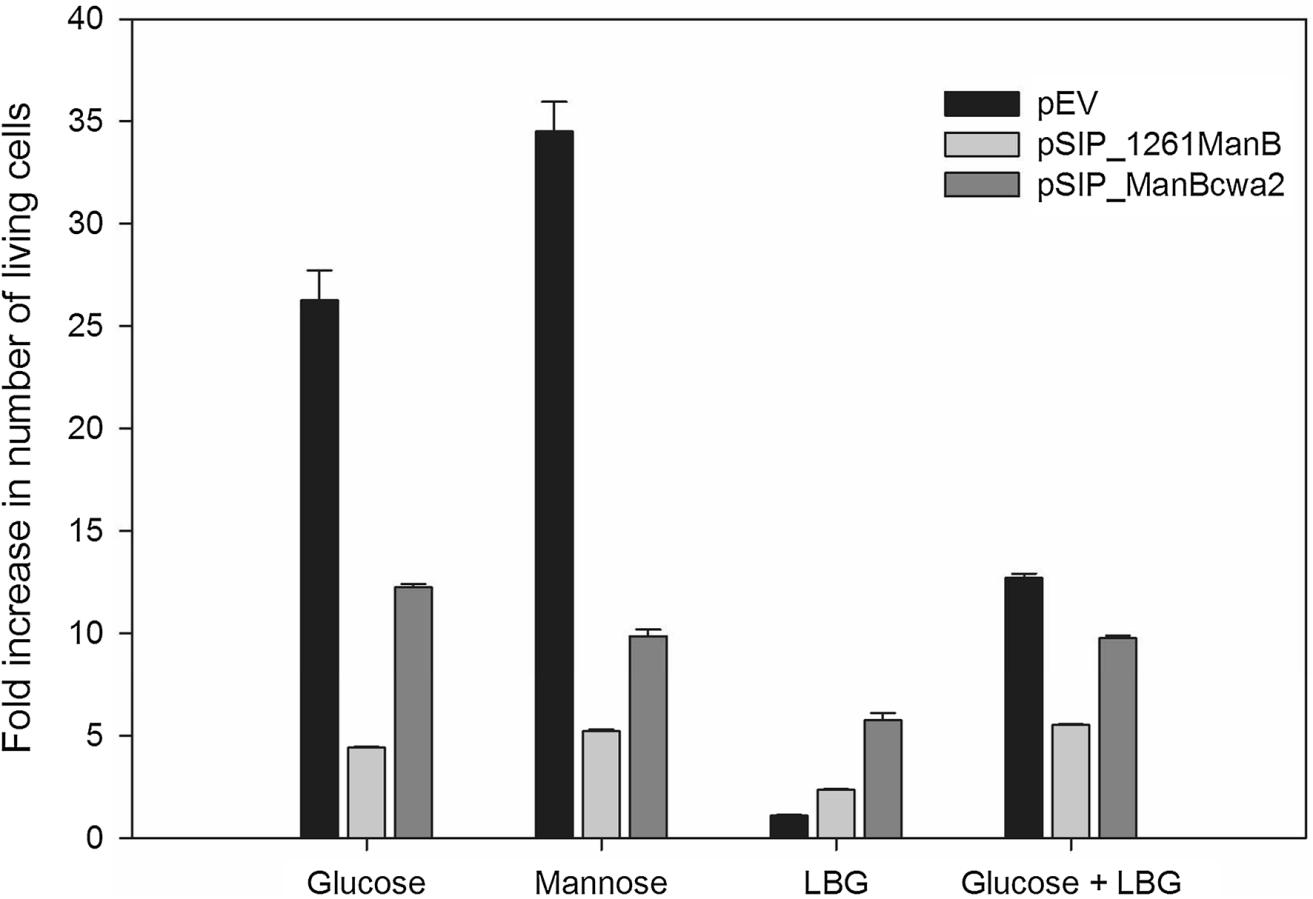
Growth of ManB-displaying *L. plantarum* cells. Growth of *L. plantarum* strains harboring plasmids designed for cell-surface display of ManB (pSIP_1261ManB and pSIP_ManBcwa2) was tested using MRS containing the indicated carbohydrates. The growth was determined after 12 h of incubation at 37 °C and the fold increase in the number of colony forming units (cfu/ml) for each culture in comparison with the growth in MRS medium without any added carbohydrate source was evaluated

To test whether bacteria having the mannanase anchored on the surface may utilize LBG as a carbon source, the bacteria were cultivated in MRS broth containing different carbohydrates (glucose, mannose, LBG, or a mixture of glucose and LBG). After 12 h of incubation at 37 °C the number of colony forming units (cfu/ml) in the cell cultures was determined. Figure 7 shows fold increase in viable cells on different media in comparison with the growth in MRS medium without any added carbohydrate source. *L. plantarum* carrying the empty vector pEV, which contains neither the ManB-encoding gene nor the signal and anchoring sequences, grew as expected very well in MRS broth containing glucose or mannose. The growth of this strain in MRS broth containing LBG was poor, which is similar to the growth in MRS medium with added carbohydrate, because this strain carries the empty vector without ManB-encoding gene, therefore LBG was not broken down into smaller manno-oligosaccharides to support its growth. When this strain is grown in MRS medium containing 1:1 mixture of glucose and LBG, the increase in the number of living cells is mainly due to the presence of glucose in the medium. The growth of both strains producing ManB was considerably lower compared to the control strain (pEV), which is a well-known characteristic of recombinant lactobacilli that overproduce heterologous proteins. This was most pronounced for the strain harboring the lipoprotein-anchor, which showed lower cell numbers on all carbohydrates tested compared to the strain with the cell wall anchor. Interestingly, in contrast to the control strain, both strains displaying ManB showed notable growth on MRS containing LBG as the sole carbohydrate. These observations show that surface-displayed ManB is able to break down galactomannan (LBG) into smaller MOS (including mannose, Fig. 5) and that these MOS can support growth of *L. plantarum.* However, the growth on MRS containing LBG as the sole carbohydrate was not comparable with the growth when glucose/mannose were included in the medium (Fig. 7) because the cells prefer monosaccharides such as mannose or glucose for their growth. When these sugars depleted in the medium, the cells will then consume other oligosaccharides for their growth.

## Discussion

Lactic acid bacteria are important microorganisms in the food and beverages industry. Over the past decades, LAB have been used not only as starter cultures, but also as producers of flavoring enzymes, antimicrobial peptides or metabolites that contribute to the flavor, texture and safety of food products [22–24]. LAB have for a long time been used in the production of a wide range of foods without adverse effects on humans [25]. Nowadays, LAB receive increasing attention because of their potential application in probiotic products [26]. Due to their food-grade status and probiotic characteristics, several LAB are considered as safe and effective cell factories for food applications [23, 24]. *Lactobacillus plantarum*, one of the best-studied LAB for this purpose, is a versatile lactic acid bacterium, which is encountered in a range of environmental niches including dairy, meat and especially vegetable fermentations. Moreover, it is commonly found in the human gastrointestinal tract. Thus, the improvement of genetic tools for efficient protein expression, secretion and cell surface display in lactobacilli is an important aspect in further development of LAB as food-grade cell factories and carriers of recombinant enzymes for food/feed applications.

The use of enzymes in food technology has found wide application. Economical, sustainable and smart use of these biocatalysts could involve immobilization, where the enzyme after extraction from the fermentation process is bound onto a suitable food-grade carrier. The immobilization process adds to the costs of the biocatalyst preparation, and hence is restricted to more costly enzymes that are used in food technology, while cheaper enzymes are only used one time and then discarded. In this study we investigated an approach that is based on the use of food-grade *L. plantarum* both as the cell factory and as carrier for enzyme immobilization. This will enable the direct use of the microbial cells straight after the fermentation step.

We explored this concept using a mannanase and a chitosanase, which are relevant for the production of health-promoting and potentially prebiotic oligosaccharides. The two anchors employed were also from *L. plantarum*, a lipoprotein-anchor derived from the Lp_1261 protein and a cell wall anchor (cwa2) derived from the Lp_2578 protein. Both anchors were successfully used to display the enzymes on the cell surface of *L. plantarum* WCFS1, but differences were observed. Flow cytometry and immunofluorescence microscopy confirmed surface localization of ManB with both anchors and of CsnA with cell wall anchor. Surface localization of CsnA with the lipoprotein anchor could not be confirmed by flow cytometry and immunofluorescence microscopy. We speculate this could be the smaller size of CsnA (~30 kDa; ManB has a mass of ~41 kDa) and the attachment of the N-terminus of the lipoprotein anchor to the plasma membrane rather than the cell wall as with cwa2. The Myc-tag of CsnA might be buried in the cell wall and thus not sufficiently exposed for recognition by the antibody used for Myc detection. However, this type of discrepancies is not an unusual observation. For example, a similar observation was previously reported for a membrane anchor (Lipobox domain, Lip) when using it to display an anti-DEC-205 antibody (aDec) at the surface of *L. plantarum* [27]. The anchored aDec was not detected at the surface with flow cytometry, however the anchored protein was found to be functional. Activity measurements showed that lipoprotein anchored CsnA is produced, but that the activity is not stably bound to the cell surface (Fig. 4b). It is thus conceivable that the lipo-anchoring of CsnA did not work as anticipated.

Anchored ManB and CsnA were able to convert LBG and chitosan to oligosaccharides. Based on the known specific activities of the purified soluble enzymes (1800 U/mg for ManB and 800 U/mg for CsnA) [28], the amounts of active surface-anchored protein using cell wall anchor system are in the range of ~0.5 mg per g dry cell weight for ManB and ~1.7 mg per g dry cell weight for CsnA. The strains producing cell wall anchored enzymes gave highest activities. It is worth to mention that no enzymatic activities were detected in the culture medium and Western blotting of the supernatants of enzymatic reactions (after separating the cells) showed no bands (data not shown) indicating that there was no release (or cell lysis) from the cells. Thus, the activities reported here are indeed from surface-anchored enzymes. Notably, it is not possible to say whether this higher activity is caused by higher efficiency in enzyme production, secretion and anchoring, or by a more exposed localization of the enzyme and hence better accessibility of the active sites.

When anchoring a chitosanase from *Paenibacillus fukuinensis* to the surface of *Saccharomyces cerevisiae* cells by fusion to the C-terminal half of α-agglutinin, 1.626 mM of free d-glucosamine were formed after 6 h of incubation with glycol chitosan [3]. Cell wall-anchored CsnA in our study produced 11.0 mM of free reducing end equivalents after 5 min of incubation with chitosan (data not shown) suggesting that chitosanase surface display in *L. plantarum* seems relatively effective. Displayed mannanase activities of 460 and 890 U/g dry cell weight, using the lipoprotein anchor and the cell wall anchor respectively, obtained are significantly higher than a reported activity of 62.3 U/g dry cell weight for a mannanase (*manI*) from *B. subtilis* HB002 that had been anchored to the cell-surface of *Yarrowia lipolytica* [29]. Although direct comparisons of the various systems is difficult, our present data do seem to indicate that display of enzymes on the *Lactobacillus* cell surface compares well to yeast-based systems.

The stability of the displayed enzyme is an important parameter for success. In this respect, our study revealed considerable differences between the various constructs. CsnA displayed using the cell wall anchor and ManB displayed using the lipoprotein anchor were relatively stable, retaining approximately 85 and 80 %, respectively, of their initial activities after four cycles of substrate hydrolysis. On the other hand, CsnA displayed using the lipoprotein anchor and ManB displayed using the cell wall anchor showed significantly lower stability. We have no proper explanation for these differences, which could related to different protease susceptibilities of the various fusion proteins and/or to variations in protein shedding. As it stands, it is not possible to make general statements as to the applicability of an anchoring motif. Different combinations of enzymes and anchors may have to be tested to obtain optimal results, and this is relative simple with the modular pSIP system.

## Conclusions

We have demonstrated successful anchoring of two different glycoside hydrolases onto the surface of *L. plantarum*. The displayed enzymes are active and some of the enzyme-producing cells showed good stability as whole cell biocatalysts. Surface anchoring of secreted enzymes in lactobacilli may yield safe, stable, food-grade whole-cell biocatalysts that can be used in different production processes relevant for the food industry. In addition, the use of lactobacilli rather than other bacterial species often used as microbial cell factories may help to overcome the reluctance of the food industry when it comes to introducing these novel biological production processes.

## Methods

### Chemicals, enzymes and plasmids

All chemicals were purchased from Sigma (St. Louis, MO, USA), unless stated otherwise, and were of the highest quality available. All restriction enzymes and corresponding buffers were purchased from New England BioLabs (Frankfurt am Main, Germany. The plasmids pSIP409-ManB-native and pSIP409-CsnA-native containing the mannanase gene *manB* from *B. licheniformis* DSM13 (ATCC 14580) or the chitosanase gene *csnA* from *B. subtilis* 168 (ATCC 23857) [28] were used as templates for amplification of these two genes, respectively. All plasmids used in this study are listed in Table 1.

**Table 1:** Strains and plasmids used in this study

Strain or plasmid	Relevant characteristic (s)	Reference source
Strains		
*L. plantarum* WCFS1	Host strain	[8]
*E. coli* TOP10	Host strain	Invitrogen
Plasmids		
pEV	Erm^r^; pLp_2578sAmyA derivative, no signal sequence, no *man* or *csn* (negative control)	[16]
pSIP409-ManB-native	Erm^r^; *spp*- based expression vector pSIP409, for expression of *manB* its native signal peptide	[28]
pSIP409-CsnA-native	Erm^r^; *spp*- based expression vector pSIP409 for expression of *csnA* with native signal peptide	[28]
pLp_1261InvS	Erm^r^; pSIP401 derivative with the lipoanchor sequence from Lp_1261 fused to part of the *inv* gene.)	[16]
pLp_3050_DC_Ag85B_E6_cwa2	Erm^r^; pSIP401 derivative encoding the Lp_3050 signal peptide ([32]) translationally fused to the hybrid antigen DC-Ag85B-ESAT6, followed by the cell wall anchor (cwa2) from Lp_2578 (cwa2 comprises 194 residues of Lp_2578; [17])	(Unpublished)
pSIP_1261ManB	Erm^r^; pLp_1261InvS derivative with *manB*-*myc* instead of the *inv* gene	This study
pSIP_1261CsnA	Erm^r^; pLp_1261InvS derivative with *csnA*-*myc* instead of the *inv* gene	This study
pSIP_ManBcwa2	Erm^r^; pLp_3050_DC_Ag85B_E6_cwa2 derivative with *manB*-*myc* instead of the gene fragment encoding DC_Ag85B-E6	This study
pSIP_CsnAcwa2	Erm^r^; pLp_3050_DC_Ag85B_E6_cwa2 derivative with *csnA*-*myc* instead of the gene fragment encoding DC_Ag85B-E6	This study

### Bacterial strains, media and culture conditions

The bacterial strains used in this study are listed in Table 1. *Lactobacillus plantarum* WCFS1, isolated from human saliva as described by Kleerebezem et al. [8], was originally obtained from NIZO Food Research (Ede, The Netherlands) and maintained in the culture collection of the Norwegian University of Life Sciences (NMBU), Ås, Norway. *Escherichia coli* TOP10 (Invitrogen; Carlsbad, CA, USA) was used in subcloning of DNA fragments and was grown in brain heart infusion (BHI) broth (Oxoid Ltd.; Basingstoke, UK) at 37 °C. *L. plantarum* was grown in deMan, Rogosa and Sharpe (MRS) broth (Oxoid) at 37 °C without agitation. When needed, erythromycin was supplemented to media to final concentrations of 200 µg/ml and 5 µg/ml for *E. coli* and *L. plantarum* cultivations, respectively.

### DNA manipulation

Plasmids were isolated from *E. coli* cells using the Nucleospin Plasmid Miniprep Kit (Macherey–Nagel; Bethlehem, PA, USA). PCR amplification of DNA was performed using Phusion high-fidelity DNA polymerase F530-S (New England BioLabs) and the primers listed in Table 2, which were purchased from Operon Biotechnologies (Cologne, Germany). The sequences of PCR-generated inserts were verified by DNA sequencing performed by a commercial provider (Microsynth; Vienna, Austria). PCR products and DNA fragments obtained by digestion with restriction enzymes were purified using the Nucleo-Spin Extract II Kit (Macherey–Nagel), and the DNA concentration was estimated using the Qubit™ dsDNA BR assay (Invitrogen). Ligation of DNA fragments was performed using T4 DNA ligase (Fermentas; Vilnius, Lithuania) and In-Fusion Cloning Kit (Clontech; Mountain View, CA, USA) following the manufacturers’ instructions. All plasmids were transformed into *E. coli* One Shot TOP10 chemically competent cells (Invitrogen) following the manufacturer’s protocol. After obtaining the plasmids in sufficient amounts, they were transformed into electrocompetent cells of *L. plantarum* WCFS1 according to the protocol of Aukrust and Blom [30].

**Table 2:** Primers used in this study

Primer	Sequence^a^ 5′→3′	Restriction site underlined
Man3050F	TGCTTCATCAGTCGAC *CACACCGTTTCTCCGGT*	*Sal*I
ManR1	TGAGATGAGTTTTTGTTC*TTCCACGACAGGCGTCAAAGAAT*	
ManR2	TCAGATCCTCTTC*TGAGATGAGTTTTTGTTCTTCCACGAC*	
3050R3	GTTCAGTGACACGCG *T* *CAGATCCTCTTCTGAGATG*	*Mlu*I
Man1261F	GATTGCGGCGGTCGAC *CACACCGTTTCTCCGGT*	*Sal*I
1261R3	CCGGGGTACCGAATTC *TTACAGATCCTCTTCTGAGATG*	*EcoR*I
Csn3050F	TGCTTCATCAGTCGAC *GCGGGACTGAATAAAGATC*	*Sal*I
CsnR1	TGAGATGAGTTTTTGTTC*GTCGACAGATCCTTTGATTAC*	
CsnR2	CAGATCCTCTTC*TGAGATGAGTTTTTGTTCGTCGACAGA*	
Csn1261F	GATTGCGGCGGTCGAC *GCGGGACTGAATAAAGATC*	*Sal*I

^a^The nucleotides in italics are the positions that anneal to the DNA of the target gene (*manB* or *cnsA*)

### Plasmid construction

A schematic overview for the construction of the expression cassette for secretion and anchoring of both ManB and CsnA is presented in Fig. 1. The two anchoring sequences used in this study were taken from pLp_1261InvS and pLp_3050_DC_Ag85B_E6_cwa2 (Table 1), which are derivatives of the pSIP401 vector that has been developed for inducible gene expression in lactobacilli [31]. The plasmids contain N-terminal signal peptides derived from the genes encoding Lp_1261 [16] and Lp_3050 [32], respectively. The total length of the Lp_1261 anchor is 75 residues, including 22 amino acids of the SP. The cell wall anchor sequence comprises 223 C-terminal residues from Lp_2578, of which 189 residues are the linker region followed by the LPQTSE motif, which is followed by a hydrophobic stretch and a positively charged C-terminal [16, 17, 33]. The C-termini of the target genes, *manB* and *csnA*, were fused to a 30-bp fragment encoding the *myc* tag (GAACAAAAACTCATCTCAGAAGAGGATCTG), as shown in Fig. 1.

For construction of pSIP_ManBcwa2 a *manB*-*myc* fragment was generated by three PCR steps: PCR1 with primers Man3050F and ManR1, PCR2 with primers Man3050F and ManR2, and PCR3 with primers Man3050F and 3050R3 to introduce a C-terminal *Mlu*I site. The resulting PCR fragment (~1047 bp) was ligated into the *Sal*I/*Mlu*I-digested vector pLp_3050_DC_Ag85B_E6_cwa2 using In-Fusion^®^ HD Cloning Kit (Clontech) yielding the plasmid pSIP_Mancwa2.

For construction of pSIP_1261ManB, the *manB*-*myc* fragment generated from the PCR2 step described above was used as the template for a PCR reaction with primers Man1261F and 1261R3. The resulting PCR fragment was ligated into the *Sal*I/*EcoR*I-digested vector pLp_1261InvS [16] using In-Fusion cloning kit (Clontech; Mountain View, CA, USA) yielding the plasmid pSIP_1261Man.

For construction of pSIP_CsnAcwa2 and pSIP_1261CsnA, a similar cloning strategy was used. The primer pairs Csn3050F/CsnR1, Csn3050F/CsnR2, and Csn3050F/3050R3 (Table 2) were used for the construction of pSIP_CsnAcwa2, whereas the primer pair Csn1261F/1261R3 (Table 2) was used to construct pSIP_1261CsnA. In-Fusion cloning kit (Clontech; Mountain View, CA, USA) was used for the ligation during the construction of pSIP_CsnAcwa2 and pSIP_1261CsnA.

### Gene expression in *Lactobacillus plantarum*

To generate the four expression strains, pSIP_ManBcwa2, pSIP_1261ManB, pSIP_CsnAcwa2 and pSIP_1261CsnA were transformed into electro-competent *L. plantarum* WCFS1 and transformants were selected on MRS agar plates containing 5 μg/ml erythromycin. For gene expression, overnight cultures of *L. plantarum* WCFS1 harboring the plasmids were diluted in 50 ml of fresh pre-warmed MRS broth containing 5 µg/ml erythromycin to an OD_600_ of ~0.1, and incubated at 37 °C without agitation to an OD_600_ of 0.3. Gene expression was then induced by adding 25 ng/ml of the peptide pheromone IP-673 [34]. Cells were harvested 2 h after induction at an OD_600_ of approximately 0.8–1.2 by centrifugation at 4000×*g* for 10 min at 4 °C, washed twice with phosphate buffered saline (PBS) containing 137 mM NaCl, 2.7 mM KCl, 2 mM KH_2_PO_4_, and 10 mM Na_2_HPO_4_ (pH 7.4), and then re-suspended in 1 ml of PBS containing 20 µl of 50 mM PMSF.

### Western blotting

The cells obtained as described above were disrupted with glass-beads (≤106 μm; Sigma) using a FastPrep-24 instrument (MP Biomedicals, Solon, OH) by shaking at speed 6.5 for 45 s. Proteins in the cell-free extracts were separated by running 10 % NuPAGE Novex Bis–Tris gels (ThermoFisher Scientific; St. Leon-Rot, Germany) following the protocol provided by the manufacturer. Subsequently, electroblotting was performed using the iBlot Dry Blotting system (Invitrogen) according to the manufacturer’s instructions. Monoclonal murine anti-Myc antibody was obtained from Invitrogen and used as recommended by the manufacturer. The protein bands were visualized by using polyclonal rabbit anti-mouse antibody conjugated with horseradish peroxidase (HRP) (Dako, Denmark) and the SuperSignal West Pico chemiluminescent substrate from Pierce (Rockford, IL, USA).

### Flow cytometry and indirect immunofluorescence microscopy

Cell staining for flow cytometry was carried out as previously described [16] with some modifications. One ml of cell culture (OD_600_ of ~0.5) was harvested 2 h after induction, and cells were resuspended in 50 µl PBS containing 1 % of BSA (PBS-B) and 0.4 µl of the monoclonal anti-Myc antibody (Invitrogen; diluted 1:5000 in PBS-B). After incubation at RT for 30 min, the cells were centrifuged at 5000×*g* for 3 min at 4 °C and washed three times with 500 µl PBS-B. The cells were subsequently incubated with 50 µl PBS-B and 0.8 µl polyclonal rabbit anti-mouse antibody (FITC conjugated; ThermoFisher Scientific) diluted 1:2500 in PBS-B for 60 min in the dark, at room temperature. After collecting the cells by centrifugation (4000×*g*, 3 min at 4 °C) and washing five times with 500 µl PBS, the stained cells were analyzed by flow cytometry using a MACSQuant analyzer (Miltenyi Biotec; Bergisch Gladbach, Germany), following the manufacturer’s instructions.

For indirect immunofluorescence microscopy, the bacterial cells stained with monoclonal anti-c-Myc antibody and goat anti-mouse Alexa Fluor® 488 (IgG H&L) (Abcam; Cambridge, UK) were visualized under a Leica TCS SP5 II confocal laser scanning microscope (Leica Microsystems; Mannheim, Germany) using a 488-nm argon laser (FITC photomultiplier tube [PMT]) and a bright field (BF) PMT for transmitted light. The fluorescence detection window was set between 505 and 550 nm and the images were acquired with a PL APO 63×/1.40 oil immersion objective.

### Enzyme activity measurements

Enzymatic activities were determined following methods described previously [3, 29, 35, 36] with some modifications. The reaction mixtures consisted of 100 µl of a suspension the enzyme-displaying cells in PBS and 900 µl of a 0.5 % (w/v) galactomannan solution (locust bean gum, LBG; Megazyme, Bray, Ireland) for mannanase activity, or 400 µl of a 0.5 % (w/v) chitosan solution (PT Biotech Surindo, Jawa Barat, Indonesia) for chitosanase activity. The galactomannan solution was prepared by dissolving LBG in 50 mM sodium citrate buffer (pH 6.0) at 50 °C for 30 min. Chitosan was completely dissolved in 1 % acetic acid at 50 °C after 30 min, and then the pH of the solution was adjusted to 5.5.

Enzyme-displaying cells were collected from the cultures 2 h after induction by centrifugation at 4000×*g* for 5 min at 4 °C. Cell pellets obtained from 100 ml culture were washed twice with PBS and re-suspended in 100 µl of PBS. The mannanase or chitosanase-displaying cells were incubated with LBG or chitosan solutions, respectively, at 37 °C with mixing at 600 rpm for 5 min. The cells were removed by centrifugation (5000×*g*, 4 °C, and 2 min) and the amount of reducing sugars released in the supernatant of the enzymatic reaction was determined by the dinitrosalicylic acid (DNS) assay. Briefly, 100 µl of the reaction supernatant were mixed with 100 µl of DNS solution, followed by heating at 99 °C for 10 min, cooling on ice for 5 min, and dilution with 800 µl de-ionized water, before measuring the absorbance at 540 nm using 1–5 µmol/ml of d-mannose or d-glucosamine as standards for the mannanase and chitosanase assay, respectively. One unit of mannanase or chitosanase activity was defined as the amount of enzyme releasing 1 µmol of reducing sugars (or reducing end equivalents) per minute under the given conditions. The reactions were done in triplicates and the standard deviations were always less than 5 %.

As an extra control, cells obtained after the incubation with substrate were washed with 500 µl with PBS and collected by centrifugation (5000×*g*, 4 °C, 3 min). The cells were then re-suspended in 100 µl of PBS and mannanase or chitosanase activities were re-measured. This procedure was repeated for four cycles of activity measurements with intermediate washing steps to evaluate the stability of ManB and CsnA-displaying cells.

### High performance anion exchange chromatography (HPAEC)

Separation of the oligosaccharides released from locust bean gum during the activity assay described above was carried out by HPAEC analysis on an ICS-3000 system from Dionex (now Thermo Scientific; Sunnivale, CA, USA) with pulsed amperometric detection. The system was equipped with a CarboPac PA-1^®^ column (2 × 250 mm) connected to a 50 mm CarboPac PA-1 guard column (Dionex). Separation of manno- or galactomanno-oligosaccharides was performed with a multi-step linear 1 M NaOAc gradient, going from 0.1 M NaOH to 0.1 M NaOH/0.1 M NaOAc over 35 min, then to 0.1 M NaOH/0.3 M NaOAc over 25 min, and finally 0.1 M NaOH/1 M NaOAc over 5 min, prior to reconditioning with 0.1 M NaOH for 9 min. Soluble manno-oligosaccharides (DP 1, 2, 3 and 6; Megazyme) were used as standards. Before analysis, the supernatants from the activity assay were centrifuged to remove any insoluble material.

Separation of the oligosaccharides released from chitosan during the activity assay described above was also carried out by HPAEC analysis. The analysis was performed with the same instrument and column as above, but the separation was achieved by isocratic elution with 25 mM NaOH for 18 min as described in [37].

### Direct infusion mass spectrometry

Direct infusion mass spectrometry was performed on a Velos Pro LTQ linear ion trap mass spectrometer (Thermo Scientific) with electrospray ionization (ESI) interface. A continuous flow of 0.2 ml/min of 1 mM formic acid/acetonitrile (30/70) and accurate 2 µl injections were supplied by an UltiMate 3000 RSLC UHPLC system (Dionex, now Thermo Scientific) directly linked to the ESI-interface without any chromatographic separation. The ESI was operated at 4 kV positive mode with sheath gas and auxiliary gas flow in the spray of 30 and 5 (arbitrary units), respectively. The ESI-probe was heated to 250 °C for better vaporization of the mobile phase. Data were collected with full scan acquisition in the mass range from 150–2000 mz for 24 s and averages of 8–9 consecutive scans were used for illustrations.

### Growth of recombinant ManB-displaying *Lactobacillus plantarum* cells on LBG

ManB-displaying *L. plantarum* cells (strains carrying either the plasmids pSIP_1261ManB or pSIP_ManBcwa2) were harvested 2 h after induction and then grown in MRS broth containing 25 ng/ml of the peptide pheromone IP-673 and 20 g/l of different carbohydrates (glucose, mannose, LBG, or a 1:1 mixture of glucose and LBG), or no added carbohydrate substrate. After 12 h of incubation at 37 °C, the number of colony forming units (cfu/ml) for each culture was determined. The fold increase in the number of living cells in MRS medium with different carbohydrates in comparison with that in MRS medium without any added carbohydrate source was evaluated.

## Abbreviations

ManB: mannanase from *Bacillus licheniformis* DSM13 (ATCC 14580)
CsnA: chitosanase from *Bacillus subtilis* ATCC 23857
